# Tuberculosis from a Dental Standpoint

**Published:** 1912-11-15

**Authors:** A. A. McClanahan

**Affiliations:** Springfield, Tenn.


### TUBERCULOSIS FROM A DENTAL STANDPOINT.

BY A. A. McCLANAHAN, D. D. S., SPRINGFIELD, TENN.

Read before Tennessee State Dental Society, June 7, 1912.

The discovery of the bacillus tuberculosis by Robert Koch in 1882 and the demonstration of its habits, properties and effects ranks as one of the greatest contributions to medical science ever made, and of inestimable value to the health and happiness of the human family. As is well known, it has revolutionized the method of treatment of tuberculosis by raising the general health standards of those infected and increasing the powers of the natural resistive agencies.

Previous to this discovery the medical profession held to the theory that the great majority of cases of tuberculosis was due to heredity, and the laity was so taught, and, consequently, one born of tuberculous parentage lived in constant dread of the disease. While it is not now considered an inherited disease, yet an enfeebled power of resistance may be inherited, or a predisposition, and this combined with early infection from environment and unhygienic methods is the cause of nearly all the so-called hereditary cases.

Thompson says: "Many striking instances are on record in which of a series of children of tuberculous parentage those removed in early infancy and reared elsewhere have grown up in health, whereas those of the same family reared at home in a tuberculous environment have fallen early victims to the disease." Thus proving that paternal transmission of tuberculosis is not probable, and that maternal transmission through the placenta if at all possible is very rare.

Now, it is not my purpose to discuss the etiology of the disease nor to go into its history at length, but to give some figures of its awful havoc in the human family and emphasize some plain facts that doubtless you all know.

Consumption is known to have existed for three thousand years; how much longer we do not know. About two hundred thousand persons die of it annually in the United States and at this date about one million American citizens are more or less incapacitated from this same cause. Tuberculosis is the greatest foe the human family has to face. Of all the wars of history, famine, pestilences, diseases or disasters, of whatever character, there is none of them that compares to the great annual loss of life caused by the microscopic tubercle bacillus. One-seventh of all persons born in civilization die of consumption. Of every three persons who die in the prime of life one is carried off by tuberculosis—cut down by this grim monster of the hosts of Death just in the height of his usefulness and efficiency; and this, by the authority of our best medical writers, from a preventable disease. Tuberculosis is the most universal disease known and is no respector of persons—neither race, age nor sex is exempt. The high and the low, the rich and the poor, the man of toil and the man of leisure, all succumb to the Great White Plague alike. Neither is it confined to certain localities or countries but is found in every country and under every government on the globe. It is true according to statistics, that some localities are more favorable to the development of tuberculosis than others as well as some occupations, and the mortality is much greater in the prime of life—between the ages of 20 and 40—but no occupation is exempt and at no age is one immune.

With this brief outline of appalling facts before us, is it not time for the dental profession to move up and assert itself in helping the physician and the anti-tuberculosis societies over the country in stamping out this disease? The time was when people believed that human mortality followed an unyielding law; that death inevitably occurred at a constant rate. But all such false ideas are fast sinking into oblivion, and more scientific knowledge is taking their place. It is now known that mortality decreases as the human family observes the laws of hygiene and that it is possible for man to rid himself of every paracitic disease.

Tuberculosis being now recognized as an infectious disease may be produced by inhalation, by being taken into the mouth with impure food, and otherwise, or by direct inoculation. What wonderful opportunities are open to the dental profession to assist in eradicating this plague from the human family. For years we were taught that infectious germs were taken into our bodies with the air. To-day we have overpowering evidence at hand showing that it is seldom the air that we breathe that carries the germs but the common-drinking-cup, the fingers, the lead-pencil, the food and insects, and all of these, except the latter class, pass into the mouth the first two or three inches of the alimentary tract,—over which the dentist should have control—where under favorable conditions they multiply in sufficient numbers as to cause infection of the entire body.

We do not wish to intimate that the dentist should undertake the therapeutic treatment of tuberculosis, but since the oral cavity is the main gateway to the entire body and it is through this gateway that a large majority of the infectious diseases human flesh is heir to enters the system, I do not believe it possible to estimate the value of oral hygiene for the prevention of tuberculosis.

Dr. Knoph, of New York, says: "The latest and the most glorious achievement of dental science is dental hygiene, for dental hygiene means prevention and preservation, and these bear the closest relationship to the prevention of tuberculosis." Further, he says: "One of the earliest and very frequent symptoms of tuberculosis is impaired digestion. While I would not wish to say that bad teeth consitute the only cause of digestive disturbance, if bad teeth are present they are a factor contributing to this pathologic conditon. Ulcerated teeth may give entrance into the bone to tubercle bacilli that have been accidentally inhaled or have been contracted by secondary infection. The modern treatment of tuberculosis consists in the judicious use of fresh air, plenty of good food, plenty of pure water inside and outside, careful regulation of rest, work and medical supervision. The proper feeding of the patient is one of the most essential factors; this, however can not be accomplished if the patient has bad teeth. Some of our largest tuberculosis hospitals and sanatoriums are without a dentist. Hundreds of the consumptive poor in our institutions should have an entirely new set of teeth because they have but a very few bad ones left and still we say to them, 'chew your food well and eat plenty.' How can we expect these patients to chew well and eat plenty and get well when they are thus handicapped in the most essential part of the treatment."

Dr. Osler writes: "There is not any one thing more important to the public in the whole range of hygiene than the hygiene of the mouth. If I were asked to say whether more physical deterioration was produced by alcohol or by defective teeth I should unhesitatingly say defective teeth."

We might give quotation after quotation from eminent men, both in the dental and medical professions, as to the great value of oral hygiene and doubtless we would all agree and yet, are we practicing what we preach? Do we really understand the intrinsic value of the subject? Do we fully comprehend the fact that the mouth reeking with filth, with a temperature of 98 to 100 degrees, decayed teeth filled with putrescent matter, pyorrhea pockets, teeth covered with tarter, and diseased gums, is one of the best incubators in the world for the propagation of pathogenic germs?

Dental bacteriology is yet in its infancy. It is now only about twenty six years since Miller began his work in Koch's laboratory which resulted in his demonstrating the active cause of caries and further establishing the fact that an unhygienic mouth is a breeding ground for disease-producing germs and is the portal through which a large portion of the infectious diseases find entrance into the body. For some years, however, after this discovery we were so zealously engaged in combating the ravages of decay that we overlooked the importance of oral hygiene as a prerequisite to good health. Hunter, of London, furnished unquestionable evidence that decayed teeth and diseased gums were incubators for the tubercle bacillus and other pathogenic germs.

Moeller, found the tubercle bacillus in three mouths out of twenty persons examined. Park, of New York, discovered an abundance of tubercular germs in the mouths of nine out of fifteen persons examined who were in good physical condition. Dr. George Cook found the tubercle bacillus in one hundred and seventy one mouths out of two hundred and twenty examined. Wm. H. DeFord says: "It has been shown that pyorrhea pockets, the folds of the cheek, the spaces between the cheeks and gums, and the uncleansed pouches formed by the buccinator muscles teem with tubercular bacilli which momentarily swim out into the saliva in the act of swallowing."

Frederick B. Morehead writes: "It invades the territory of every medical specialist without apology, and yet the mouth is probably the most serious factor in the matter of infection from this organism."

In the face of such evidence is it not time we as dentists were waking up to our duty in helping wipe out the worst physical foe to the human family? Were it not for the power of resistance which nature has so wisely provided we all sooner or later would be compelled to yield up the battle and go the way of the other one-seventh of the population of the earth.

The length to which this paper would be drawn, will not permit me to go into a discussion of methods to accomplish the ends desired. That is sufficient for another paper. My purpose is to impress the important place we occupy in the world's effort to reduce mortality from tuberculosis.

Standing as we do the guardians of the very portal of human existence—the mouth—we are in a position to be regarded by our efforts as at least the third in importance of the battling triumvirate—the physician, the anti-tuberculosis league, and the dentist,—making war on humanity's ever-active and powerful enemy, the Great White Plague.

Our study and our teaching of hygienic methods is a potent factor in placing us before the public no longer as mechanics but professional men—men of scientific attainment. In proportion as we render service to humanity will it place us to the forefront as its benefactors. Our foremost physicians and dentists of to-day are those who have contributed to science preventive methods. The discovery of the germ theory of disease has placed a limitless importance on the profession of dentistry. The dentist comes in contact with humanity while it is yet in apparent active health. The physician is more often summoned after the germ has got in its work. Here is the dentist's field for teaching. Here is his opportunity for increasing the usefulness of his profession.

Science is constantly in the race with our complex modern life—the one to lengthen mortality, the other to lower it. Within recent years science has put a few years on the average. I believe that we have it within our power as dentists by studying, teaching and practicing still more advanced methods to increase the span of life still further.

DISCUSSION.

Dr. E. W. Lundy: While the president has called upon me to open this discussion, I feel that I am wholly unable to do so.

The subject is one that we ought to know more about than we do. We come in contact with it every day, and the worst of it is, when we meet it, we do not recognize it. Doubtless numbers of cases of so called Tuberculosis in a great many instances would be under control to a greater extent, if we only knew.

I have for the first time been made to fully realize just how important it was to be careful in the treatment of the mouth along oral surgery lines. I call to my mind at present a case in which the Doctor held out slight hope for the recovery of the patient, one on whom I had spent several hours trying to discover the cause, and at the time, symptoms of tuberculosis were present, and this is the disease, of which some investigation should be made in the mouth to recognize tuberculosis.

I agree with Doctor that it behooves us to take upon ourselves a new interest in this special line of work. If we should confine ourselves more fully to particular investigations, much more so to those who look well, in that when we take charge of a patient to see that his mouth is as clean as we can have it before we proceed to make other operations. It is a rule in my practice, first in attempting to relieve patients from conditions, to clean the mouth and not only clean it myself, but put him on the job himself; that is providing them with all the toilet washes that are necessary.

So long as the germs that are associated with this disease appear, a few things as to their characteristics should be known to all dentists. We should become so thoroughly familiar with the nature of the disease that we could readily suspect it the moment we come in contact with it. I expect from this time on to take more interest in this particular line of work in my practice.

Dr. Ewell Neal: This is one subject in which I am deeply interested. I am ashamed that some members of our profession are acknowledging that they are not entitled to be called Doctors of Dental Surgery. They are only dentists working upon teeth. This is a disgrace to our profession and is the reason the medical Doctors fail to give us the title we pretend to possess. We owe it to the human family to make it a practice to do what we can to prevent the spread of this dreadful disease.

How many dentists would be able to look at a patient and diagnose a case of tuberculosis? How many of us would know the bacillus if we should look at it through the microscope? We are demanding the title of a healing specialist that we do not deserve, but the name dentist is the place for us. Instead of clamoring for the name and title of doctor we should strive to live up to its significance and bring our title up to a level where it belongs. We must know how to protect our patients from possible contagion through our hands. It is an absolute disgrace that dentists are allowed to practice who can not detect this disease on sight by conditions of the mouth which indicate its presence in the patient, so that he may take proper cautions to avoid injury to other patients. Those who have not taken the trouble to examine certain of your patients should buy a microscope, if you have not access to one, and do a little work along this line. It will be interesting, to say the least, and I am confident you will gain profitable knowledge of this most important subject.

DR. D. M. CATTELL: I want to say to the author of the paper, that I am here as a living example of just such comments as he makes in his paper.

My uncles, some of my cousins, and my sister, all died of tuberculosis, and I had the same tendency. People prognosticated tuberculosis every time I had a cough, they put on long faces, and said "He's going just like his cousins; others would say, "Good Bye to David, he's going just like the rest of his family." They looked at me with long faces and tears in their eyes, and said to each other, "The next time you hear from David, he's dead." I went home with that old cough; there was my sister, brother, my uncle and aunts, still looking at me with that long face, "David is going just like the rest." I said, "I shall not die of tuburculosis." I packed my trunk and immediately changed my place of residence and recovered. I believe in some cases if we could get the patient's mind diverted by removal to some other climate and from the notion that they had to die of tuberculosis, you would find that there would not be so many deaths from the disease. I arose simply to confirm what the author of the paper has said of the importance of an early rather than a late diagnosis. Conditions in America are such that I just wanted to stand before you and show you that what I have been fighting against and what a dentist can do for others as well as himself, if he will only get a little the start of this disease.

Dr. Richards: I want to stand up for the dental profession from two standpoints. We have had quite a little abuse from our own brother here. I want the author of the paper at least to know that the Medical Profession is just about as careless or ignorant in this matter as we are. I was in a hospital recently where there was a child about ten years old who had been operated on for tuberculosis of the jaw, and the surgeon made a complete operation from his standpoint.

I was only visiting the hospital with a friend who called my attention to this little girl and was consulted in regard to her teeth. I cannot criticise what has been done here. She is a tuberculosis patient, and she needs something besides an operation. There was an opening through the jaw and the child was walking through the ward spitting through the hole, she was just as incapable of retaining the saliva in her mouth, as a baby. The whole front of her apron was wet down to her knees. I said, why don't you close that hole and prevent the discharge. She asked how would I stop it? Make her a little bib or oval bib, with a neck and pocket, pin it down, and as fast as they get wet burn them. We will do that she said. I examined the child's mouth and found an alveolar abscess which I advised and endeavored to have removed. The child had been left in that condition without a suggestion from any one as to how they might prevent that source of infection, because it was not thought important.

You all are aware of the recent article from London condemning American dentistry. I am frank to say the head of our nation need not try to defend us in this case for we have really got just what we deserved on that point.

We should not be expected to know all or how much disease there is prevailing. But we should take notice and follow these suggestions and let them influence our professional conduct and then we will be entitled to be called Doctors. I want to say however that the Medical Profession is not without its deficiencies.

